# Repeatable Perming via Thiol–Michael Click Reaction: Using Amide Derived from Maleic Acid and Cystine

**DOI:** 10.3390/molecules31020382

**Published:** 2026-01-21

**Authors:** Zezhi Liu, Ling Ma, Timson Chen, Zhizhen Li, Ya Chen, Jinhua Li, Kuan Chang, Jing Wang

**Affiliations:** 1 School of Chemical and Material Engineering (School of Cosmetics), Jiangnan University, Wuxi 214122, China; 6230606078@stu.jiangnan.edu.cn (Z.L.); jinhua.li@jnmwht.com (J.L.); 2 Guangzhou Aogu Cosmetics Manufacturing Co., Ltd., Guangzhou 510810, China; chendiansong@adolph.cn (T.C.); lizhizhen@adolph.cn (Z.L.); chenya@haservey.com (Y.C.); 3 Jiangnan Institute of Beauty Research, Wuxi 214112, China

**Keywords:** hair perming, disulfide bond, oxidative damage, hair keratin fiber, thiol–Michael

## Abstract

Conventional perming relies on oxidative agents that significantly damage hair. The thiol–Michael click perming strategy derived from linear aliphatic diols and diamines has been developed to avoid oxidative damage, but lacks repeatable perming capabilities. In this study, a novel thiol–Michael click perming molecule was proposed for repeatable perming while avoiding oxidative damage. *N*,*N*′-bis(maleoyl)-l-cystine (MA2-CySS) was synthesized and characterized through Raman spectroscopy and ^1^H NMR with MTT assay demonstrated no cytotoxicity up to 1000 μg/mL. Click reactivity analysis revealed that the reaction reached a plateau after 30 min, with alkaline pH and elevated temperatures significantly enhancing reactivity. MA2-CySS perming achieved efficiency comparable to oxidative perming, exceeding 1300% across three perming cycles. MA2-CySS perming significantly reduced both color change and cuticle damage, as demonstrated by color difference measurements and SEM, while maintaining superior mechanical properties as revealed by tensile property tests. Raman spectroscopy demonstrated that MA2-CySS perming better preserves hair keratin’s secondary structure, maintaining superior *α*-helix content at 27.50% versus 24.35%, exhibiting higher disulfide bond retention at 85% versus 72%, and showing gauche–gauche–gauche to trans–gauche–trans conformational conversion at 9% versus 6%. This study demonstrates that repeatable perming via thiol–Michael click reaction represents a significant advancement in perming methodology.

## 1. Introduction

Since the development of oxidative perming in the 1940s, hair perming has become increasingly convenient and sophisticated for individuals seeking hair styling options [1]. Conventional oxidative perming can be fundamentally characterized as a two-step process comprising reduction followed by oxidation. Initially, reducing agents like thioglycolate and cysteine cleave disulfide bonds (-S-S-) in hair keratin, converting them to thiol (-SH). Subsequently, oxidizing agents including H_2_O_2_ and NaBrO_3_ facilitate the reformation of -S-S- in new positions, resulting in the desired hairstyle. This reduction–oxidation cycle allows for repeated perming to change hairstyles, which is crucial for the widespread adoption of perming techniques [2].

However, oxidative perming agents can cause severe damage to the hair’s lipid layer and melanin, resulting in lusterless hair, discoloration, and other adverse effects. It is noteworthy that increased hair brittleness was reported after perming because oxidative perming agents cause excessive oxidation of -SH and -S-S- to sulfonate (-${SO}_{3}^{-}$), reducing -S-S- content. Importantly, -S-S- contribute much to the shape, stability, and texture of the hair. Consequently, oxidative perming causes a decrease in the α-helix content of keratin, thereby increasing brittleness. Moreover, due to their strong irritating properties, improper application or residues of oxidative perming agents can harm the scalp. To avoid these problems, it is essential to find a hair-friendly and non-irritating alternative to replace oxidative agents in hair perming formulations [3,4,5,6].

Significant research efforts have been devoted toward developing novel technologies that prevent oxidative damage during hair perming. These primarily follow two strategies, and the first is the reconstruction of -S-S- in hair using peptides or proteins which contain -SH. Cruz et al. [7] utilized synthesized keratin peptides containing -SH derived from the human genome to straighten curly hair. Fan et al. [8] used bioinspired peptides containing -SH which were designed through bioinformatics and expressed in engineered *E. coli* to create permanent hair styling effects. However, the widespread application of these peptides remains limited due to the complexity of their synthesis, expression, and purification processes. Moreover, their perming performance was slightly lower compared to oxidative perming. Tinoco et al. [9] successfully demonstrated that keratins obtained through reductive methods can serve as novel hair styling agents, but the perming efficiency was still lower than oxidative perming. Furthermore, these studies lack exploration in perming repeatability. The second perming strategy is converting -SH in reduced hair to alternative covalent bonds. Song et al. [10] used non-toxic polycarboxylic acids in hair perming, generating thioester bonds between the -SH in reduced hair and polycarboxylic acids at 180 °C, thereby enabling effective hair styling. Fang et al. [11,12] employed Fe^2+^ from metal polyphenolic networks to crosslink with the -SH in reduced hair via -Fe-S-, thereby enabling simultaneous hair dyeing and perming. Although these methods demonstrate perming performances comparable to oxidative perming, the strategy involving conversion of -S-S- to alternative forms prevents perming repeatability and may exhibit properties substantially different from virgin hair.

Utilizing the click chemistry properties of -SH in hair offers a novel strategy for developing advanced hair styling agents. In our previous investigations, a series of compounds incorporating α,β-unsaturated carbonyls derived from linear aliphatic diols and diamines were developed. Thiol–Michael click chemistry successfully facilitated the perming process under daily usage scenarios while effectively avoiding oxidative hair damage. However, its further application is limited by a lack of perming repeatability [13,14].

In this study, *N*,*N*′-bis(maleoyl)-l-cystine (MA2-CySS) was successfully synthesized and its cytotoxicity assayed. The click reactivity with -SH in hair was examined across various durations, pH values, temperatures, and concentration levels. The capability for repeatable perming was compared to conventional oxidative perming. The influences of perming on hair color, surface properties and tensile properties were analyzed using a fiber-optic spectrometer, SEM, and tensile property tests, respectively. Raman spectroscopy was employed to investigate the internal structural changes in permed hair, including the proportions of keratin secondary conformations, residual -S-S- percentage, and -S-S- conformations.

## 2. Results and Discussion

### 2.1. Synthesis and Characterization of MA2-CySS

**Figure 1:**
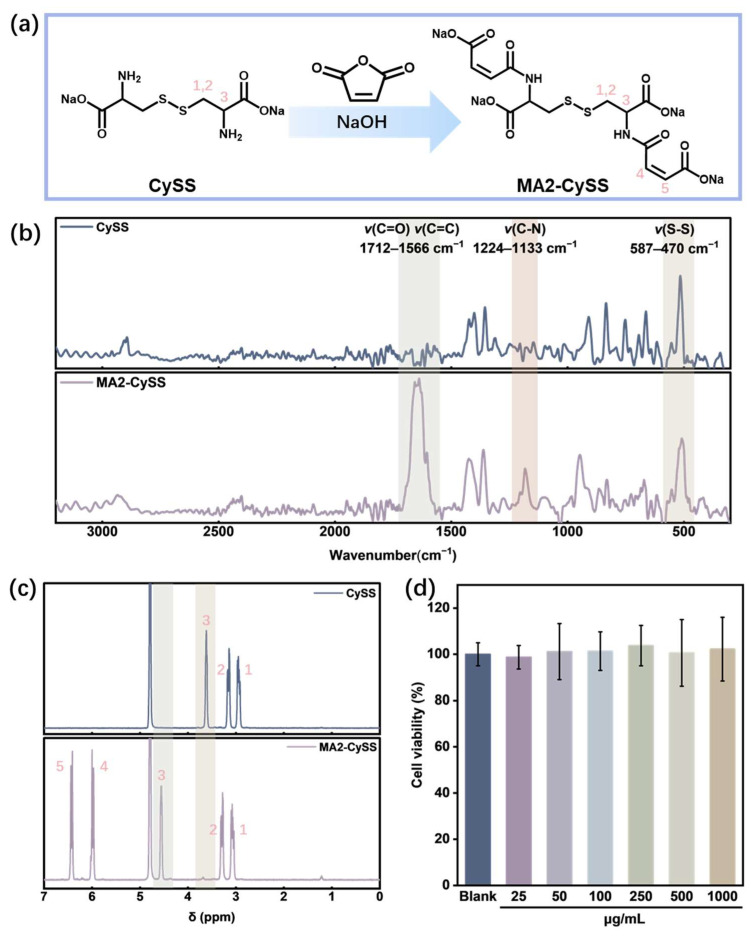
The synthesis and characterization of MA2-CySS. (**a**) The synthesis route of MA2-CySS; (**b**) Raman spectrum of CySS and MA2-CySS; (**c**) ^1^H NMR of CySS and MA2-CySS (numbers 1–5 denote the positions of H atoms); (**d**) cytotoxicity of MA2-CySS.

MA2-CySS was prepared according to Section 3.2., appearing as a yellow powder (Figure 1a). The reaction proceeded rapidly and was considered complete upon stabilization of the pH. The retention times of the starting material and products with different molar ratios were characterized by High-Performance Liquid Chromatography (HPLC). As shown in Figure S1 (Supplementary Materials), the reaction at a molar ratio of n_(CySS)_:n_(MA)_ = 1:2 yielded MA2-CySS with the highest purity. Calculated from its relative peak area in HPLC, the yield reached 96.00%. Raman spectroscopy was employed to verify the molecular structure of MA2-CySS. As shown in Figure 1b, comparative analysis of the Raman spectra between l-Cystine (CySS) and MA2-CySS revealed that both compounds exhibited characteristic -S-S- peaks at 587–470 cm^−1^, indicating the presence of -S-S- in both structures. The MA2-CySS exhibited a prominent broad absorption band at 1712–1566 cm^−1^, which was attributed to the overlapping spectral contributions from the *ν*(C=O) of the amide I band and the *ν*(C=C) of the maleoyl. The presence of the *ν*(C-N) at 1182 cm^−1^ further confirms that the product was successfully synthesized via amide bond formation while preserving the -S-S-. The ^1^H NMR spectrum (Figure 1c and Table S1) clearly shows the chemical shift (δ) change of the H at position 3, which is consistent with amino amidation, confirming the molecular structure. Quantitative integration of this signal yielded an acylation degree of 98.04%. The results obtained using the Time-of-Flight Mass Spectrometer (TOF MS, Figure S2) show that the most abundant peak at *m*/*z* 435.0177 corresponds to the deprotonated ion [M − H]^−^. The deviation between the measured value (435.0177) and the theoretical value (435.0168) is only 0.0009 Da. These results conclusively demonstrate the successful synthesis of MA2-CySS.

Considering the application of MA2-CySS on hair and scalp, its cytotoxicity was evaluated through MTT assay on HaCaT cells. The results shown in Figure 1d indicated that MA2-CySS exhibited no cytotoxicity at concentrations up to 1000 μg/mL. Compared to oxidative perming, which is known to cause scalp irritation [15,16,17], this result suggests the potential advantage of MA2-CySS in perming applications. Nevertheless, systematic toxicological and irritation studies will be essential for its future practical use.

### 2.2. Click Reactivity with -SH in Hair

**Figure 2:**
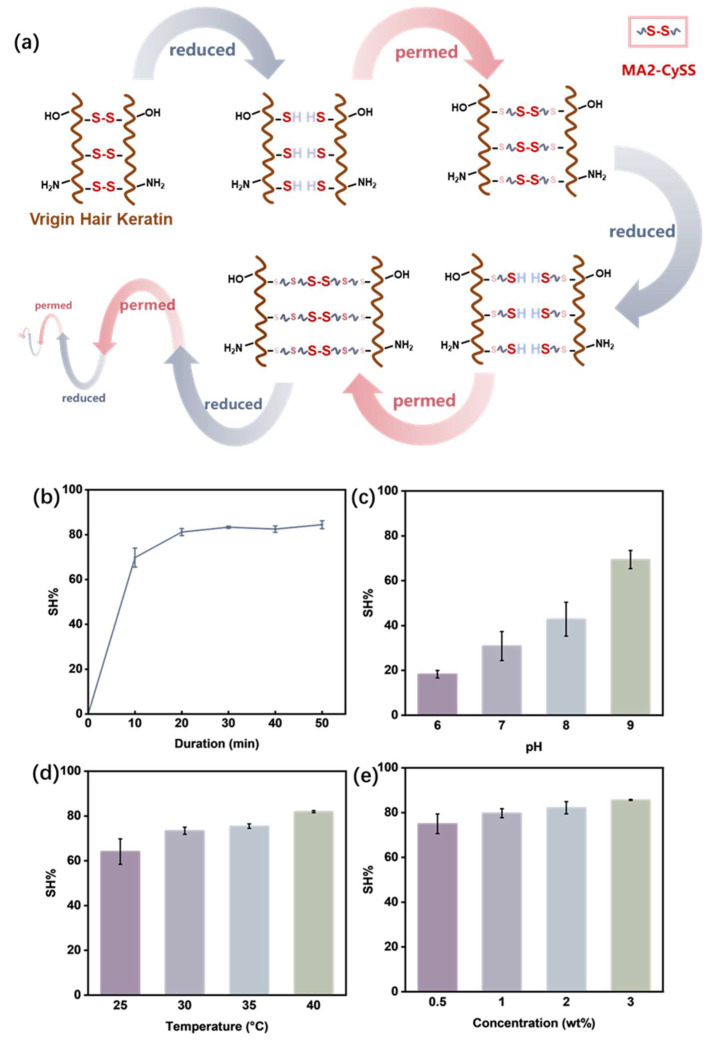
(**a**) MA2-CySS repeatable perming mechanism. Effects on click reactivity; (**b**) reaction kinetics (pH 9, 40 °C, 2 wt%); (**c**) pH (ambient, 2 wt%, 40 min); (**d**) temperature (pH 9, 2 wt%, 40 min); (**e**) concentration (pH 9, 40 °C, 40 min); *n* = 3.

The mechanism of MA2-CySS repeatable perming is illustrated in Figure 2a. The maleoyl in MA2-CySS undergoes a thiol–Michael click reaction with -SH in keratin, which forms -S-C-. Simultaneously, intramolecular -S-S- of MA2-CySS supplements the -S-S- in hair. MA2-CySS ensures the preservation of -S-S- following perming, thereby conferring repeatable perming capability to permed hair. Additionally, MA2-CySS allows keratin to maintain its structural integrity through -S-S- rather than alternative forms, thereby maximizing the consistency of properties between virgin and permed hair. Importantly, the supplement of -S-S- was expected to contribute to the stability and texture of the hair, as it can maintain the ordered conformation of keratin structures, and can hold tensile properties of permed hair.

Based on the scheme, determining the optimal click reactivity is crucial for the application of MA2-CySS in perming. The percentage decrease in -SH content (SH%) was used to evaluate the click reaction reactivity, with the detailed calculation method described in Section 3.4. The click reactivity with -SH in hair was examined across various durations, pH values, temperatures, and concentration levels. Kinetic analysis (Figure 2b) revealed that the reaction reached a plateau after 30 min. The effect of pH on click efficiency is shown in Figure 2c. Increasing pH from 6 to 9 elevated SH% from 18.31% to 69.41%, demonstrating that the reaction proceeded much more efficiently under alkaline conditions. This indicated that in aqueous media, the -SH groups of reduced hair are deprotonated to form -S^−^, which then nucleophilically attacks the -C=C- bonds to form -C-S- [18,19,20]. As illustrated in Figure 2d, temperature significantly influenced the click efficiency, where an increase from 25 to 40 °C raised SH% from 64.11% to 81.92%. In comparison, as shown in Figure 2e, the concentration of MA2-CySS had minimal effect on SH%. The SH% was only elevated from 75.01% to 85.65% when the concentration increased from 0.5 wt% to 3 wt%. In conclusion, the optimal click reactivity with -SH in hair for the perming process using MA2-CySS was determined as pH 9, 2 wt%, 40 °C for 30 min, under which SH% reached approximately 82%.

Regarding hair, it is true that basic amino acid residues such as lysine in hair can also undergo an aza-Michael addition reaction with maleoyl groups, achieving an effect similar to the thiol–Michael click reaction [21,22]. However, due to the overwhelming selectivity advantage of the thiol–Michael reaction over the aza-Michael reaction in terms of kinetics and reaction progression [23,24], and considering that the content of cysteine in hair (17.00 mol%) is nearly 6.54 times that of lysine (2.6 mol%) [25], the study focuses on the thiol–Michael reaction while neglecting the aza-Michael addition for reasons of selectivity, molar ratio, and model simplification.

While basic amino acid residues in hair (e.g., lysine) can also undergo aza-Michael addition with maleoyl groups, producing an effect analogous to the thiol–Michael click reaction [21,22], based on the overwhelming selectivity advantage of the thiol–Michael reaction over the aza-Michael reaction in terms of kinetics and reaction progression [23,24], and considering that the content of cysteine in hair (17.00 mol%) is nearly 6.54 times that of lysine (2.6 mol%) [25], as well as for the purposes of model simplification, the aza-Michael reaction was not considered.

### 2.3. Repeatable Perming Performance

**Figure 3:**
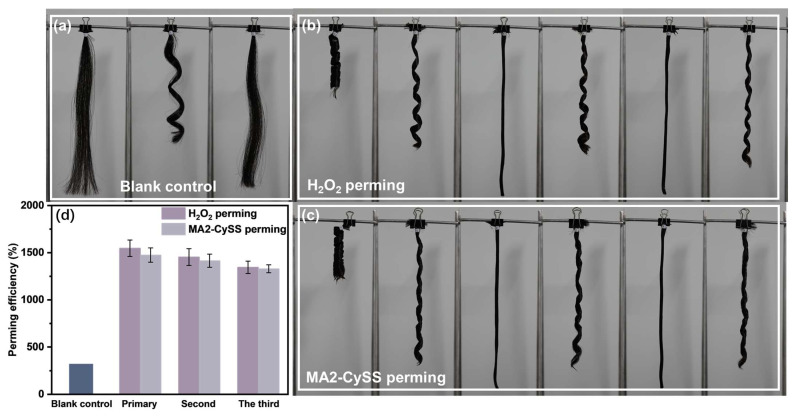
Pictures of permed hair: (**a**) Blank control, (**b**) H_2_O_2_ perming, (**c**) MA2-CySS perming, (**d**) perming efficiency.

The repeatable perming capability of MA2-CySS was evaluated through repeated perming cycles. As illustrated in Figure 3a, Blank control exhibited virtually no perming effect after hydrogen bond disruption while both MA2-CySS perming and H_2_O_2_ perming maintained curl retention. Following perming cycles with MA2-CySS and H_2_O_2_, Figure 3b,c shows that the hair successfully regained its curl pattern, providing compelling evidence that both H_2_O_2_-oxidized and reconstructed -S-S- and MA2-CySS-introduced -S-S- can undergo repeated cleavage and reformation cycles. A quantitative comparison of perming efficiency of the two methods is shown in Figure 3d. It was revealed that MA2-CySS perming demonstrated comparable repeatable perming performance to H_2_O_2_ perming, with efficiency exceeding 1300% across three perming cycles.

Although repeatable perming performance was validated under controlled laboratory conditions, it must be acknowledged that immediate perming cycles cannot fully replicate the diverse challenges encountered in daily consumer use. Factors such as washing, heat styling, and UV exposure, which may affect perm longevity, were not evaluated in this study. Future work should develop more comprehensive and consumer-relevant methodologies for assessing repeatable perming performance and perm durability.

### 2.4. Effects of Different Perming Methods on Hair Properties

Instances of hair discoloration, roughness, and brittleness after oxidative perming have been widely documented. To assess the effects of MA2-CySS perming on hair properties, a comprehensive analysis was conducted using color difference (Δ*E*) measurements, SEM characterization, and tensile property testing.

**Figure 4:**
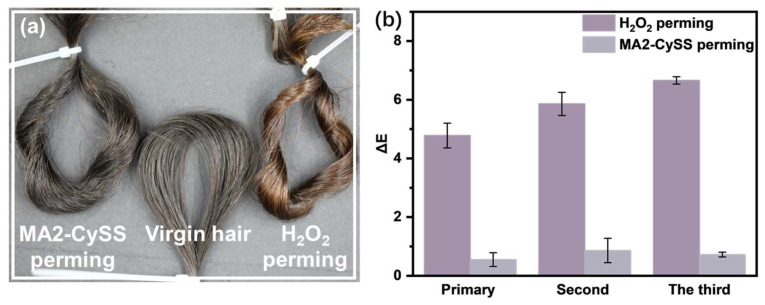
(**a**) Appearance of different hair samples. (**b**) Δ*E* of perming hair.

The changes in hair color during perming were indicated by Δ*E*. As shown in Figure 4a,b, H_2_O_2_ perming exhibited a color shift toward brown, with Δ*E* increasing significantly with cycles of perming. This color change occurred because H_2_O_2_ oxidized and damages the melanin [3]. In contrast, the MA2-CySS group presented consistently low Δ*E*, with hair retaining its original black color even after three perming cycles. This evidence strongly suggests that MA2-CySS perming offers superior hair melanin protection compared to oxidative perming.

**Figure 5:**
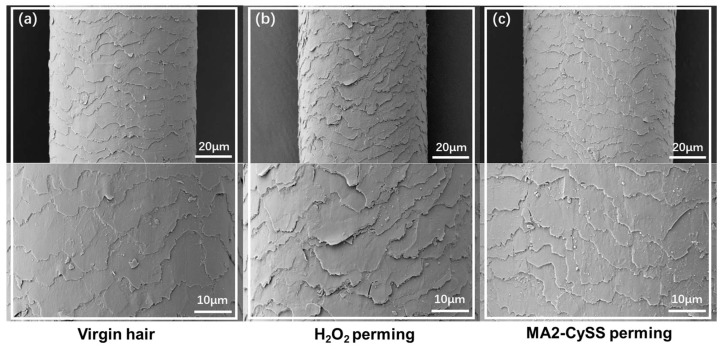
SEM images of (**a**) virgin hair, (**b**) H_2_O_2_ perming hair, and (**c**) MA2-CySS perming hair.

The surface alterations of hair cuticles were investigated using SEM. Compared to virgin hair, it was clearly observed that damage was caused by H_2_O_2_ perming (Figure 5a,b). The cuticle scales no longer adhered tightly to the hair shaft, showing severe lifting with extensive cracking. In contrast, MA2-CySS perming exhibited minimal surface alterations (Figure 5c), with only small areas of lift. The cuticle scales remained predominantly tightly adhered and the overall hair stayed intact. The observed damage is consistent with the studies on pH-induced hair changes hair by Malinauskyte, E. et al. [26] and Adav, S.S. et al. [27], indicating that the damage is not directly caused by MA2-CySS, but is attribute to the alkaline environment required for MA2-CySS perming. Consistent with our previous research, comparative analysis confirms that MA2-CySS perming causes significantly less damage to the hair surface, while better preserving hair color integrity than H_2_O_2_ perming, suggesting that the thiol–Michael click perming strategy effectively avoids oxidative damage. However, it is also necessary to acknowledge that the thiol–Michael click perming strategy has certain limitations. A healthy human scalp has a pH of 5.4–5.9, while the pH of the hair shaft is naturally mildly acidic between pH 4.5 and pH 5.5 [27]. Therefore, optimizing the applicable pH range of this strategy to better align with the hair’s natural acidic environment will be an important direction for our future work in perming methodology.

**Figure 6:**
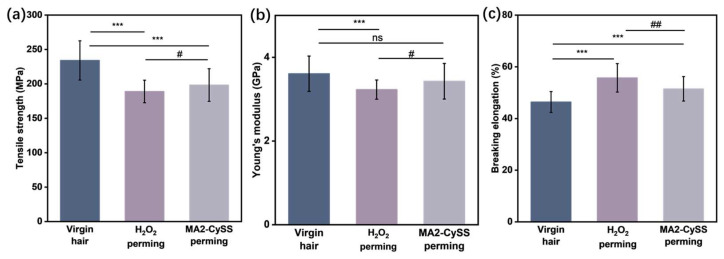
(**a**) Tensile strength, (**b**) Young’s modulus, and (**c**) breaking elongation of hair samples (# *p* < 0.05; ## *p* < 0.01; *** *p* < 0.001; ns: not significant; *n* = 30).

Changes in tensile properties of hair provide consumers with one of the most immediate perceptions of hair state. These properties are typically assessed through tensile strength (*σ*), Young’s modulus (*E*), and breaking elongation (*ε*), the detailed calculations for which are provided in Section 3.8. The results are presented in Figure 6a–c, showing values for virgin hair (*σ* = 234.10 MPa, *E* = 3.61 GPa, *ε* = 46.38%), H_2_O_2_ perming (*σ* = 189.00 MPa, *E* = 3.23 GPa, *ε* = 55.75%), and MA2-CySS perming (*σ* = 198.40 MPa, *E* = 3.43 GPa, *ε* = 51.48%). The results indicate that hair exhibited properties deviating from virgin hair after three cycles, specifically showing decreased *σ* and *E*, as well as increased *ε*. Comparative H_2_O_2_ perming analysis revealed MA2-CySS perming had significantly higher *σ* and *E* while exhibiting notably lower *ε*, suggesting MA2-CySS perming is more hair-friendly than conventional oxidative perming.

### 2.5. Effects on -S-S- in Hair Structure

**Figure 7:**
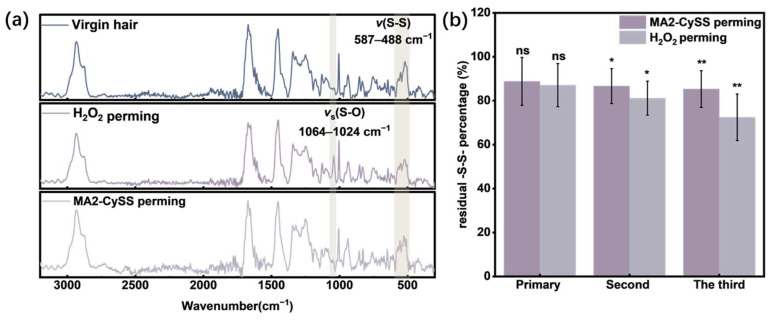
(**a**) Raman spectra of perming hair. (**b**) The residual -S-S- percentage of perming hair (* indicates the *t* test statistical significance compared to virgin hair, * *p* < 0.05, ** 0.001< *p* < 0.01, ns = not significant, *n* = 5).

The characterization of hair structure after perming cycles was conducted using Raman spectroscopy, as shown in Figure 7a. The hair samples after three H_2_O_2_ perming cycles showed a peak at 1040 cm^−1^ corresponding to the *νₛ*(S-O) vibration of -${SO}_{3}^{-}.$ This indicates that conventional oxidative perming excessively oxidized -SH and -S-S- to form -${SO}_{3}^{-}.$ [5]. In contrast, hair samples that were subjected to three MA2-CySS perming cycles showed no *νₛ*(S-O) peak, which is consistent with virgin hair, demonstrating superior ability of MA2-CySS perming to avoid -${SO}_{3}^{-}$ formation.

The retention rate of -S-S- significantly influences tensile properties of hair and maintains keratin secondary conformation [28,29]. In this study, it is a critical indicator for evaluating the capacity of repeated perming. Analysis of Raman spectroscopy is shown in Figure 7b, demonstrating that -S-S- was effectively maintained above 85% after three MA2-CySS perming cycles. The residual -S-S- percentage decreased significantly to 72% in H_2_O_2_ perming hair with increasing perming cycles, resulting from excessive oxidation converting both -SH and -S-S- into -${SO}_{3}^{-}$.

**Figure 8:**
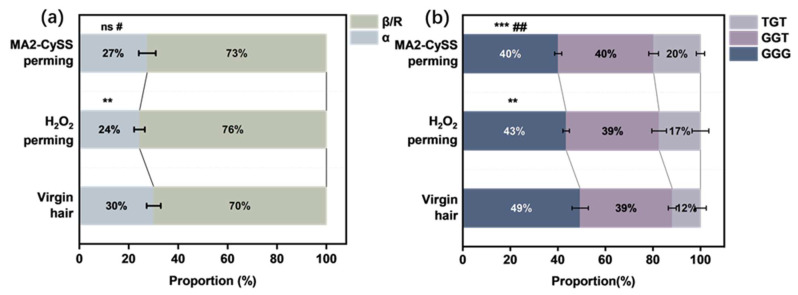
(**a**) The proportion of α and β/R conformational content. (**b**) The proportion of -S-S- conformational content (*/# indicates the *t* test statistical significance compared to virgin hair/H_2_O_2_ perming hair, # *p* < 0.05, **/## 0.001< *p* < 0.01, *** *p* < 0.001, ns = not significant, *n* = 5).

Moreover, ordered keratin configurations especially for α-helix play essential roles in maintaining hair properties, particularly its tensile properties [30,31,32]. Through spectral simulation of the Amide I band, the proportions of keratin secondary conformation after three perming cycles were calculated and are shown in Figure 8a. These results reveal that the α content of hair permed with H_2_O_2_ decreased significantly from 30.07% to 24.35%. This result provides a good explanation for the tensile properties, and it can be inferred that structural transition of keratin from *α* to *β*/*R* was induced by excessive oxidation converting both -SH and -S-S- into -${SO}_{3}^{-}$ by H_2_O_2_. This structural transformation resulted in decreased *σ* and increased *ε*. In contrast, hair permed with MA2-CySS maintained a considerably higher proportion of *α* at 27.50% compared to H_2_O_2_ permed hairs. Comprehensive analysis indicates that sufficient -S-S- retention after perming with MA2-CySS maintained the ordered conformation of keratin structures, thereby preserving the structural integrity of the hair.

The influence of -S-S- conformation on hair tensile properties remains underexplored. While Schlücker et al. [29] observed an increased proportion of gauche–gauche–trans (GGT) and trans–gauche–trans (TGT) conformations in brittle hair from trichothiodystrophy patients, such hair also shows a 50% reduction in -S-S- content. Thus, brittleness cannot be attributed solely to conformational change. In our study, curve-fitting of the -S-S- band (Figure 8b) revealed that both MA2-CySS and H_2_O_2_ perming reduced gauche–gauche–gauche (GGG) and increased TGT content, showing a more pronounced shift of around 9% in MA2-CySS perming. This is likely due to MA2-CySS bridging intra-/interchain -SH groups, extending the bond toward the TGT conformation [29,33]. Despite H_2_O_2_ perming retaining a higher GGG proportion, it exhibited lower overall -S-S- content and inferior tensile performance compared to MA2-CySS perming. Therefore, compared to conformational change, maintaining high -S-S- content is the primary determinant of tensile strength. This highlights the practical need to preserve disulfide bonds in perming technology development.

## 3. Materials and Methods

### 3.1. Reagents and Materials

Sodium (2*R*,2′*R*)-3,3′-disulfanediylbis(2-Aminopropanoate) (CySS, ≥99%), maleic anhydride (MA, ≥99%), 3-(4,5-dimethylthiazol-2-yl)-2,5-diphenyltetrazolium bromide (MTT, ≥99%), sodium dodecyl sulfate (SDS, ≥99%), thioglycolic acid (TGA, ≥95%), edetate disodium dihydrate (EDTA, ≥99%), 5,5′-dithiobis(2-nitrobenzoic acid) (DTNB, ≥98%) were procured from Adamas Reagent Co., Ltd. (Shanghai, China). Hydrogen peroxide (H_2_O_2_, 30 wt%) was procured from Sinopharm Chemical Reagent Co., Ltd. (Shanghai, China). HaCaT cells were provided by BeNa Culture Collection (Shanghai, China). Other reagents in cell experiments were purchased from Grand Island Biological Company (Los Angeles, CA, USA). Virgin white Chinese hair (22 cm × 1 g, free 20 cm) and virgin black Chinese hair (22 cm × 1 g, free 20 cm) were purchased from Shanghai Canyu commercial Co., Ltd. (Shanghai, China). All hair samples were cleaned with 10 wt% SDS before use.

### 3.2. Synthesis and Characterization of MA2-CySS

MA2-CySS was synthesized through N-amidation reaction between MA and CySS. Typically, 0.035 mol CySS was dissolved in 100 g of water and stirred in an ice bath. Then, 0.07 mol MA was added, while 20 wt% NaOH was added dropwise to maintain the pH at approximately 10.5 throughout the reaction. Upon completion, the mixture was freeze-dried to obtain the solid product, which was subsequently purified by ethanol washing.

The molecular structure of MA2-CySS was confirmed using Raman spectroscopy, ^1^H NMR spectroscopy, and TOF MS. Raman spectra (inVia Reflex, Renishaw, Gloucestershire, UK) were collected in the range of 300–3200 cm^−1^ with an excitation wavelength of 785 nm. ^1^H NMR spectra (AVANCE III HD 400 MHz, Bruker, Billerica, MA, USA) were acquired using D_2_O as the solvent. TOF MS (MALDI SYNAPT MS, Waters, MA, USA) were recorded in negative electrospray ionization mode, using 0.01 mg/mL aqueous solution of MA2-CySS. The purity of the products was assessed by HPLC, which included analysis of the starting material and the products obtained at different molar ratios.

### 3.3. MTT Assay for Cytotoxicity

The cytotoxicity of MA2-CySS was evaluated using MTT assay. HaCaT cells were seeded in 96-well plates (1 × 10^5^ cells/mL, 100 µL per well) and incubated with various concentrations of MA2-CySS for 24 h. Each concentration was tested in six replicate wells. After the treatment period, 100 µL of MTT solution (0.5 mg/mL) was added to each well, followed by incubation for an additional 4 h at 37 °C. Subsequently, the culture medium was carefully aspirated, and the formed formazan crystals were solubilized by adding 100 µL of DMSO to each well. The absorbance was measured at 490 nm using a microplate reader (Tecan Infinite 200Pro, Thermo Fisher Scientific, Massachusetts, USA) [34]. Cell viability was calculated according to the following formula:

Cell viability (%) = *OD_T_*/*OD_B_* × 100% (1)

where *OD_T_* and *OD_B_* represent the average *OD* of the experimental group and the blank group, respectively.

### 3.4. Determination of Click Reactivity with -SH in Hair

Click reactivity was determined by measuring the decreased -SH content using Ellman reagent, identifying optimal click reaction using MA2-CySS [35]. Virgin hair samples were immersed in an 8 wt% TGA with pH 8.0 at 40 °C for 30 min. Then, the samples were immersed in MA2-CySS solution under various experimental conditions including pH, temperature, concentration and duration. Hair samples were retrieved and washed, then chopped and suspended in 1.0 mL of 0.1 M TBS buffer with pH 8.0 containing 0.4 mg/mL DTNB. After being incubated for 20 min, the absorbance of supernatant at 412 nm was measured using a microplate reader. The reduced hair samples without MA2-CySS treatment served as the reference. The percentage decrease in -SH content (*SH*%) was calculated using following equation:

*SH*% = (*A_R_*/*m_R_* − *A*/*m*)/(*A_R_*/*m_R_* − *A*_0_/*m*_0_) × 100% (2)

where *SH%* represents percentage decrease in -SH content, *A* and *m* are the absorbance and mass of the tested hair sample, *A_R_* and *m_R_* are the absorbance and mass of the reduced reference hair sample, and *A_0_* and *m_0_* are the absorbance and mass of virgin hair, respectively.

### 3.5. Evaluation of Perming Repeatability

The perming repeatability was quantified by perming efficiency. Reduced hair was obtained by the same procedure as Section 2.4. Then, the hair samples were secured onto plastic perm rods and immersed in a 2 wt% MA2-CySS with pH 9.0 at 40 °C for 30 min. Upon completion, the samples were dried under controlled conditions at 25 ± 2 °C and 50 ± 5% RH, then carefully removed from the perm rods and rinsed in deionized water for 5 min to allow natural uncoiling to disrupt hydrogen bonds. After drying, the hair length was precisely measured and documented photographically for subsequent analysis. This entire cycle was repeated three times to simulate repeated perming. The positive control was designed to simulate conventional oxidative perming. A 3 wt% H_2_O_2_ at pH 3.0 was selected for this purpose, based on our earlier work indicating that H_2_O_2_ and NaBrO_3_ produce comparable perming performance and oxidative damage. Deionized water was used as a blank control to demonstrate no perming effect [14,36]. Perming efficiency was calculated according to the formula:

Perming efficiency (%) = *(L_0_*/*N_0_*)/(*L*/*N*) × 100% (3)

where *L_0_* and *N_0_* are length and loop number before perming, *L* and *N* are length and loop number after perming, respectively.

### 3.6. Color Difference

Color difference (Δ*E*) of hair during the perming was quantified using a fiber-optic spectrometer (LS173, Linshang, Shenzhen, China), with values automatically calculated by the instrument. Δ*E* measurements were repeated five times on different regions of each hair sample [37].

### 3.7. Scanning Electron Microscopy (SEM)

SEM (SEM5000X, China-Guoyi Quantum, Hefei, China) was utilized to characterize the surface morphology of virgin hair and perming hair after three perming cycles. Hair samples were affixed to aluminum stubs via conductive carbon tape and subsequently sputter-coated with gold to ensure sufficient electrical conductivity. Observations were conducted at 5 kV, with images acquired at appropriate magnifications to visualize surface features.

### 3.8. Tensile Property Tests

Tensile properties of virgin hair and perming hair after three perming cycles were evaluated using a fiber strength tester (XS (08) XT-3, Xusai Instrument, Shanghai, China). For each group of hair strands, 30 single hair fibers with diameters of 70–90 μm were selected from the strands using a laser rangefinder (LSM-6200, Mitutoyo, Yokohama, Japan). All tests were carried out under controlled conditions at 25 ± 2 °C and 50 ± 5% RH [38]. The tensile strength (*σ*), Young’s modulus (*E*), and breaking elongation (*ε*) of the single hair fiber were calculated using the following formula:

*σ* = *F_b_*/*S* (4)

*E* = (*F_H_*/*S*)/(Δ*L_H_*/*L*_0_) (5)

*ε* = Δ*L_b_*/*L*_0_ (6)

where *F_b_*, *F_H_*, *S*, Δ*L_b_*, Δ*L_H_* and *L_0_* are breaking strength, Hooke’s strength, cross-sectional area of hair fiber, breaking displacement, Hooke’s displacement, and initial length, respectively.

### 3.9. Raman Spectroscopy

Chemical changes in hair were analyzed using a Raman microscope spectrometer. Raman spectra were collected from the hair surface in the spectral range of 300–3200 cm^−1^ using an excitation wavelength of 785 nm.

The -S-S- content in hair samples was quantified by calculating the ratio between the -S-S- band peak area from 490 to 570 cm^−1^ and the -C-H band peak area from 1380 to 1500 cm^−1^. The -S-S- content of permed hair was normalized to the virgin hair and expressed as residual -S-S- percentage [39].

Spectral simulation of the Amide I band region was performed to quantify the proportion of the *β*-sheet and/or random coil forms (*β*/*R*) as well as *α*-helix form (*α*) in the secondary conformation of hair sample keratin. The conformational components were assigned with *β*/*R* at 1671 cm^−1^ and *α* at 1652 cm^−1^. The relative content of each conformational state was calculated as the percentage of its respective peak area relative to the sum of *β*/*R* and *α* peak areas [30].

Curve-fitting of the -S-S- band region was performed to quantify the proportions of different -S-S- conformations. The conformational components of -S-S- stretching were assigned to gauche–gauche–gauche (GGG) at 508 cm^−1^, gauche–gauche–trans (GGT) at 524 cm^−1^, and trans-gauche-trans (TGT) at 544 cm^−1^. The relative content of each conformational state was calculated as the percentage of its respective peak area relative to the total integrated -S-S- band area [33].

## 4. Conclusions

A novel thiol–Michael click perming molecule, MA2-CySS, has been designed and synthesized and could supplement the -S-S- in hair during perming. Repeatable perming performance of MA2-CySS was comparable to conventional oxidative perming, while MA2-CySS perming resulted in less color difference, reduced cuticle damage, and higher tensile properties of hair. Raman spectra revealed remarkable efficacy of MA2-CySS perming in avoiding excessive oxidation and maintaining high -S-S- content, which preserved the keratin secondary structure integrity, thereby ensuring better tensile properties of hair. The results demonstrate that repeatable perming via the thiol–Michael click reaction is more hair-friendly and non-irritating, with great application potential in hair care products.

## Data Availability

Data are contained within the article.

## Supplementary Materials

The following supporting information can be downloaded at https://www.mdpi.com/article/10.3390/molecules31020382/s1, Figure S1: HPLC of starting material and the products obtained at different molar ratios (nMA:nCySS); Figure S2: TOF MS of MA2-CySS; Table S1: 1H NMR spectrum of CySS and MA2-CySS.
