# Supplementary material for: Repeatable Perming via Thiol–Michael Click Reaction: Using Amide Derived from Maleic Acid and Cystine
Source: Molecules. 2026 Jan 21;31(2):382. doi: 10.3390/molecules31020382 (PMC12844010; doi:10.3390/molecules31020382)
Supplement: Supplementary file 1 [file molecules-31-00382-s001.zip › molecules-4086374-supplementary.pdf]

molecules

Supplementary Material

Repeatable Perming via Thiol–Michael Click Reaction: Using Amide Derived from Maleic Acid and Cystine

Zezhi Liu [1], Ling Ma [1,2,*], Timson Chen [2], Zhizhen Li [2], Ya Chen [2], Jinhua Li [1,3], Kuan Chang [1,3,*] and Jing Wang [1,3,*]

1 School of Chemical and Material Engineering (School of Cosmetics), Jiangnan University, Wuxi 214122, China; 6230606078@stu.jiangnan.edu.cn (Z.L.); jinhua.li@jnmwht.com (J.L.)
2 Guangzhou Aogu Cosmetics Manufacturing Co., Ltd., Guangzhou 510810, China; chendiansong@adolph.cn (T.C.); lizhizhen@adolph.cn (Z.L.); chenya@haservey.com (Y.C.)
3 Jiangnan Institute of Beauty Research, Wuxi 214112, China
* Correspondence: maling@adolph.cn (L.M.); changkuan@jiangnan.edu.cn (K.C.); jingwang@jiangnan.edu.cn (J.W.)

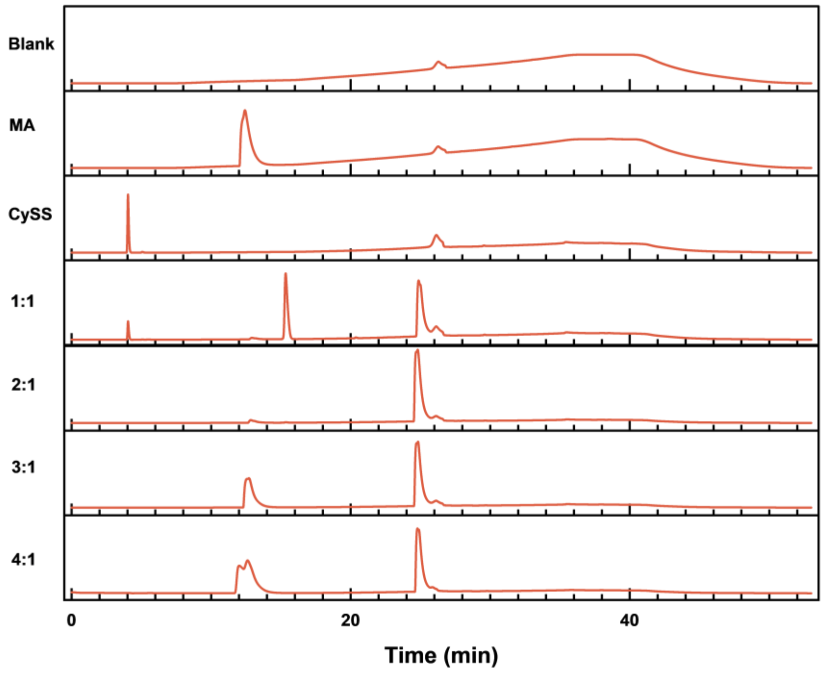

Figure S1. HPLC of starting material and the products obtained at different molar ratios (n_{MA} : n_{CySS}).

Table S1. ^{1}H NMR spectrum of CySS and MA2-CySS.

Position	δ (Cyss)	Integration (Cyss)	Peak Pattern (Cyss)	δ (MA2-Cyss)	Integration (MA2-Cyss)	Peak Pattern (MA2-Cyss)
1	2.93	2.00	q	3.08	2.00	q
2	3.14	2.00	q	3.27	2.00	q
3	3.62	2.00	t	4.56	2.00	q
4				6.00	2.00	d
5				6.41	1.95	d

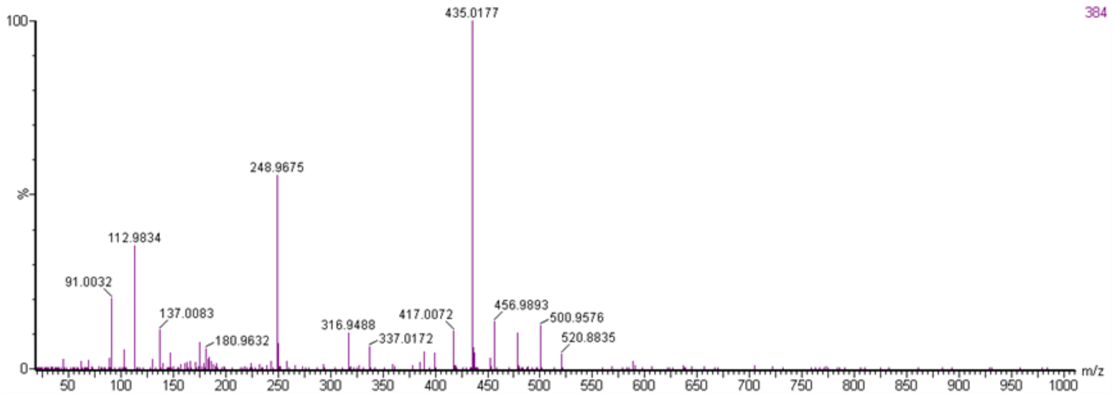

Figure S2. TOF MS of MA2-CySS.
